# Role of mitochondria in rescuing glycolytically inhibited subpopulation of triple negative but not hormone-responsive breast cancer cells

**DOI:** 10.1038/s41598-019-50141-z

**Published:** 2019-09-24

**Authors:** Asmaa Reda, Alaa Refaat, Ahmed A. Abd-Rabou, Ali Mokhtar Mahmoud, Mohamed Adel, Salwa Sabet, Sameh Saad Ali

**Affiliations:** 1 Center for Aging and Associated Diseases, Helmy Institute of Medical Sciences, Zewail City of Science and Technology, Giza, Egypt; 2 Molecular and cellular Biology department, Faculty of Science, Benha University, Benha, Egypt; 3 Scientific Research Center, Misr International University, Cairo, Egypt; 4 Molecular and Cellular Signaling division, Department of Biochemistry and Molecular Biology, Kobe University Graduate School of Medicine, Kobe, Japan; 5 Hormones Department, Medical Research Division, National Research Centre, 12622 Giza, Egypt; 6 National Research Council of Italy, Institute of Biomolecular Chemistry, Pozzuoli, Italy; 7 Department of Neuroscience, Brandeis University, Massachusetts, USA; 8 Zoology Department, Faculty of Science, Cairo University, Giza, Egypt; 9 Tumor Biology Research Program, Children’s Cancer Hospital Egypt, 57357 P.O Box 11441, 1 Seket Al-Emam Street, Cairo, Egypt

**Keywords:** Breast cancer, Energy metabolism

## Abstract

Triple-negative breast cancer (TNBC) subtype is among the most aggressive cancers with the worst prognosis and least therapeutic targetability while being more likely to spread and recur. Cancer transformations profoundly alter cellular metabolism by increasing glucose consumption *via* glycolysis to support tumorigenesis. Here we confirm that relative to ER-positive cells (MCF7), TNBC cells (MBA-MD-231) rely more on glycolysis thus providing a rationale to target these cells with glycolytic inhibitors. Indeed, iodoacetate (IA), an effective GAPDH inhibitor, caused about 70% drop in MDA-MB-231 cell viability at 20 μM while 40 μM IA was needed to decrease MCF7 cell viability only by 30% within 4 hours of treatment. However, the triple negative cells showed strong ability to recover after 24 h whereas MCF7 cells were completely eliminated at concentrations <10 μM. To understand the mechanism of MDA-MB-231 cell survival, we studied metabolic modulations associated with acute and extended treatment with IA. The resilient TNBC cell population showed a significantly greater count of cells with active mitochondria, lower apoptotic markers, normal cell cycle regulations, moderately lowered ROS, but increased mRNA levels of p27 and PARP1; all compatible with enhanced cell survival. Our results highlight an interplay between PARP and mitochondrial oxidative phosphorylation in TNBC that comes into play in response to glycolytic disruption. In the light of these findings, we suggest that combined treatment with PARP and mitochondrial inhibitors may provide novel therapeutic strategy against TNBC.

## Introduction

Breast cancer represents a major cause of death among women worldwide with more than a million new cases and hundreds of thousands deaths each year^1^. Triple-negative breast cancer (TNBC) constitutes around 20% of invasive breast cancers and is characterized by the lack of estrogen (ER) and progesterone (PR) receptors’ expression and non-amplified human epidermal growth factor receptor 2 (HER2) expression. TNBCs are diagnosed by immunohistochemistry but are late to detect and characterized by poorer therapeutic outcomes compared to other breast cancer subtypes^2^. Clinical features of TNBCs include a peak in recurrence risk within the first 3 years, a weak correlation between tumor size and lymph node metastasis, and a peak of cancer-related death in the first 5 years^3^. In spite of extensive research efforts, no targeted therapies are available to date and TNBCs are currently treated with cytotoxic combination chemotherapy. Ironically, TNBC patients respond effectively to chemotherapeutic interventions, however, these patients suffer from decreased overall survival rates^4^. An understanding of the cellular and molecular mechanisms responsible for the aggressive pattern of recurrence in TNBCs is thus crucial for the identification of targeted therapeutic strategies for this devastating disease.

Relatively recently, there is a growing interest in the bioenergetics of cancer cells^5^, with increasing needs for a better understanding of energy metabolism during cancer progression^6^. Breast cancer cells show profound bioenergetics, histological and genetic difference compared to the normal ones^7,8^. These modifications are accompanied by an unlimited cell proliferation, accelerated growth, altered metabolism, and apoptosis evasion. An altered metabolic phenotype known as Warburg effect has been described in cancer cells where an intense increase in glucose uptake through enhancement of glycolytic activity and dramatic lactate production even when oxygen supply is not scarce. This was also consistent with diminished mitochondrial respiration despite the presence of high oxygen concentration^9^. Warburg effect or aerobic glycolysis is driven in these cells by hypoxia, oncogenic stimuli, mitochondrial defects, and aberrantly enhanced expression of glycolytic enzymes^10,11^. The Warburg phenomena confers a selective survival advantage for cancer cells by using the short glycolytic path thus providing a surplus of ATP molecules. It was reported that, when compared to hormone responsive cells, TNBC cells exhibit metabolic characteristics manifested by high glycolytic activity and low mitochondrial oxidative phosphorylation (OXPHOS)^12^. We hypothesized that such metabolic phenotype in TNBC cells may render them highly sensitive to glycolytic inhibition thus opening a window for metabolic interventions specifically targeting TNBCs.

Iodoacetate is reported as a potent inhibitor of glycolysis. It mainly inhibits glyceraldehyde-3-phosphate dehydrogenase (GAPDH) enzyme, a pivotal enzyme in the pyruvate-lactate and glucose metabolism, illustrating its functional role in both mitochondrial oxidative phosphorylation and glycolysis^13–15^. Meanwhile, GAPDH enzyme catalyzes the conversion of glyceraldehyde-3-phosphate into 1,3-bisphosphoglycerate with the reduction of NAD^+^ to NADH (H^+^)^16^. Thus, GAPDH enzyme is suggested as a targetable glycolytic enzyme in the increasingly promising metabolic interventional anticancer therapy^17^. Aptly, in 1966 the pre-treatment of cancer cells with iodoacetate was reported to suppress their ability to induce tumors, and in some cases to reverse established solid tumor through inducing tumor immunity^18^. Moreover, iodoacetate reacts with the sulfhydryl (-SH) group of the cysteine residues at the active site of GAPDH to block the formation of thiohemiacetal^19^. Strikingly, being strong sulfhydryl alkylating agent, iodoacetate is anticipated to strongly modulate cellular redox homeostasis eliciting cytotoxicity^20^. Furthermore, iodoacetate is also known as an inhibitor of 6-phosphate dehydrogenase (G6PDH) and 6-phosphogluconate dehydrogenase, which are pivotal enzymes in the pentose phosphate pathway that supplies reducing energy to cells^21^. Taken together, these criteria encouraged us to explore and revive the potential of iodoacetate as a simple and inexpensive drug candidate for specific targeting of breast malignancies.

## Materials and Methods

### Cell lines

The human breast cancer cell line MDA-MB-231(CRM-HTB-26) was purchased from the American Type Culture Collection (USA) and MCF-7 was a kind gift from Dr. Sherif El-Khamisy’s laboratory at Zewail City of Science and Technology.

### Cell culture

Human breast cancer cell lines MDA-MB-231 and MCF-7 were grown and maintained in culture with Dulbecco’s Modified Eagle Medium (DMEM-F12). This media is supplemented with 10% FBS “fetal bovine serum” and 1% Penicillin Streptomycin (Pen Strep). Cells were maintained at 37 °C in a 5% CO_2_ atmosphere^22,23^.

### Cytotoxicity assay using WST-1

Cells were seeded in 96-well plates at a density of 1 × 10^4^ cells/well and allowed to settle overnight in 5% CO_2_ at 37 °C. The medium was discarded and was replaced with a fresh one containing different final concentrations of iodoacetate (0–50 μM) in triplicates and incubation was continued for 4 h and 24 h. At the end of the incubation period, the cells were rinsed with PBS and treated with 10 μl of the cell proliferation assay reagent WST-1 (Sigma-Aldrich) for 4 h. The amount of formazan dye formed as a result of the cleavage of the stable tetrazolium salt WST-1 was measured spectrophotometrically at λ = 450 nm using a FLUOstar Omega microplate reader (BMG Labtech, Germany) and is directly correlated to the cytotoxic effect of the drug.

### Metabolic profiling

The key parameters of glycolytic function including basal glycolysis, glycolytic capacity, and glycolytic reserve, were assessed using a Seahorse XF glycolysis assay (Agilent Technologies, Berlin, Germany) according to the manufacturer’s instructions. Prior to the assay, XF sensor cartridges were hydrated. To each well of an XF utility plate, 1 mL of Seahorse Bioscience calibrant was added and the XF sensor cartridges were placed on top of the utility plate, and kept at 37 °C incubator without CO_2_ for 12 hrs. MCF7 or MDA-MB-231 cells were cultured in XF24-well cell culture microplates (Agilent) at a density of 4 × 10^4^ cells/per well and then incubated for 24 h at 37 °C under 5% CO_2_ atmosphere. The cells were incubated with iodoacetate (5, 10, 15, 20 µM) for instantaneous, 4 h or 12 h pre-incubation. Oxygen consumption rate (OCR) and the extracellular acidification rate (ECAR) were measured simultaneously for 16 min to establish a baseline measurement^24^. Glycolysis, glycolytic capacity, and glycolytic reserve were calculated by subtracting the average rates before and after the addition of glucose (10 mM), ATP synthase inhibitor oligomycin (1.0 µM) and 2-deoxy-D-glucose (2-DG) (50 μM). These three compounds were injected consecutively with a specific time gap and ECAR values were measured after each injection.

Different parameters of respiration, basal respiration, coupling efficiency, and spare respiratory capacity, were investigated by using a Seahorse XF-24 cell Mito Stress assay according to the manufacturer’s instructions. The following electron transport inhibitors were added; oligomycin (1.0 μM), FCCP (0.5 μM) and a mixture of antimycin-A (0.5 μM) and rotenone (0.5 μM). The different parameters of respiration were calculated by subtracting the average respiration rates before and after the addition of the electron transport inhibitors The calculated parameters included: basal respiration (baseline respiration minus antimycin-A post injection respiration), ATP turnover (baseline respiration minus oligomycin post injection respiration), maximal respiratory capacity (FCCP stimulated respiration minus antimycin-A post injection respiration) and reserve respiratory capacity (FCCP stimulated respiration minus baseline respiration)^25^.

### Measurement of mitochondria cellular respiration

The assessment of mitochondrial oxygen consumption was analyzed using the high-resolution respirometry O2k system (Oroboros Oxygraph, Innsbruck, Austria). Briefly, 2.5 × 10^6^ cells suspended in 2 mL of mitochondrial respiration medium (MiR05: 110 mM sucrose, 0.5 mM EGTA, 3.0 mM MgCl_2_, 80 mM KCl, 60 mM K-lactobionate, 10 mM KH_2_PO_4_, 20 mM Taurine, 20 mM Hepes, 1.0 g/l BSA, pH 7.1) were added in each chamber of the system. The assessment of mitochondrial oxygen consumption started with basal respiration in which respiration is fueled by endogenous substrates. Then iodoacetate at 20 µM was added by Hamilton syringes into a 2 mL Oxygraph chamber and left for 15 min while the other chamber was used for the control untreated cells. 2 mM of malate and 10 mM of glutamate which are the substrates of complex I that is used for assessing the LEAK-I state were added. Then, for the determination the OXPHOS-I, 5 mM of ADP was added. Furthermore, to characterize OXPHOS-II, 0.5 mM of Rotenone, followed by 10 mM of succinate, which are the substrates of complex II, were added to each chamber. In addition, to inhibit complex III, 2.5 µM of Antimycin A was added. Finally, for the assessment of complex IV, 0.5 mM of TMPD and 2 mM of Ascorbate were added.

### Assessment of cell death type by flowcytometry

Normal, early apoptotic, late apoptotic and necrotic cells were distinguished by fluorescein isothiocyanate-conjugated Annexin V and by the fluorescent dye propidium iodide (PI). Briefly, cells were seeded at density 2 × 10^5^ cells/well and then the cells were treated by different doses of iodoacetate for 4–24 hrs. After that, the cells were harvested, washed with PBS, and then suspended in annexin binding buffer. Cells were stained with the Annexin V and/or Propidium iodide at room temperature. After that, cells were washed in binding buffer then the fluorescence was measured by Attune® Acoustic Focusing Cytometer.

### Cell cycle analysis by flowcytometry

Iodoacetate treatment at concentrations (5, 10, 15, 20 µM) was added in a medium for 4 and 12 hrs to MCF7 and MDA-MB-231. After that, cells were harvested, washed with PBS, fixed with ethanol. Then G0/G1, S, G2/M phases of cell cycle were evaluated by measuring the cellular uptake of PI in PBS containing RNAse (Sigma-Aldrich) using flow cytometry instrument (Attune® Acoustic Focusing Cytometer, Life Technologies).

### Quantitative real-time reverse transcription-PCR

RNA was extracted from cells using (RNeasy mini kit, Qiagen) according to the manufacturer’s manual, then cDNA was synthesized from 1 μg RNA using the High-capacity cDNA Reverse Transcription kit (Applied Biosystems). Quantitative real-time PCR (qPCR) reactions for *Bax*, *Bak*, *PARP*, *caspase 9*, *p21* and *p27* genes were performed using primer-specific annealing temperature. For SYBR GREEN-based quantitative real-time PCR reactions, each 12.5 µL reaction contained 0.4 µM primer pairs,  100 ng cDNA, 6.25 µL SYBR GREEN, and  up to 3 µL ddH_2_O. PowerUp™ SYBR™ Green Master Mix (Applied Biosystems) was used to carry out  qPCR on QuantStudio Real-Time PCR (QuantStudio 12 K Flex Real-Time PCR System). Levels of RNA were normalized to GAPDH levels and estimated as delta-delta threshold cycle (ΔΔCT). The following primers were used; Bax: Fwd-5′ GACGGCCTCCTCTCCTACTT 3′, Rev-5′ TAAGAAAAATGCCCACGTCC 3′, BAK: Fwd- 5′ GAAAAATGGCTTCGGGGCAA 3′, Rev-5′ CTGCGGAAAACCTCCTCTGT 3′, PARP: Fwd- 5′ GCCCTAAAGGCTCAGAACGA 3′, Rev- 5′CTACTCGGTCCAAGATCGCC 3′, P21: Fwd- 5′GCAGACCAGCATGACAGATTT 3′, Rev- 5′GGATTAGGGCTTCCTCTTGGA3′, P27: Fwd- 5′ ATCACAAACCCCTAGAGGGCA3′, Rev- 5′ GGGTCTGTAGTAGAACTCGGG3′. The amplification program comprised two stages, with an initial 95 °C Taq activation stage for 10 min followed by 40 cycles of 95 °C denaturation for 15 s and annealing at 60 °C for 30 s and elongation at 72 °C for 30 s. After amplification, a melting curve analysis was performed by collecting fluorescence data. GAPDH was chosen as an internal control. All samples were performed in triplicates and the relative amount of target gene was calculated using the” 2^−∆∆CT^” method.

### Analyses of intracellular reactive oxygen species and mitochondrial membrane potential by flowcytometry

Quantification of intracellular reactive oxygen species (ROS) was performed using 2′,7′-dichlorodihydrofluorescein diacetate (DCF-DA, Sigma), a non-fluorescent dye, which is de-esterified intracellularly and turns to highly fluorescent form by intracellular ROS, as per manufacturer’s manual. MCF7 and MDA-MB-231 cells were treated with different concentrations of iodoacetate (5, 10, 15 and 20 µM) against control untreated cells. Assessment of mitochondrial transmembrane potential (ΔΨ_m_) was performed using TMRE dye. Cells were co-stained with 1 µM DCF and 500 nM TMRE. 20,000 events per replicate was collected and then mean and median fluorescence were quantified.

## Results

### Metabolic phenotyping of breast cancer cells and effects of iodoacetate

**Figure 1:**
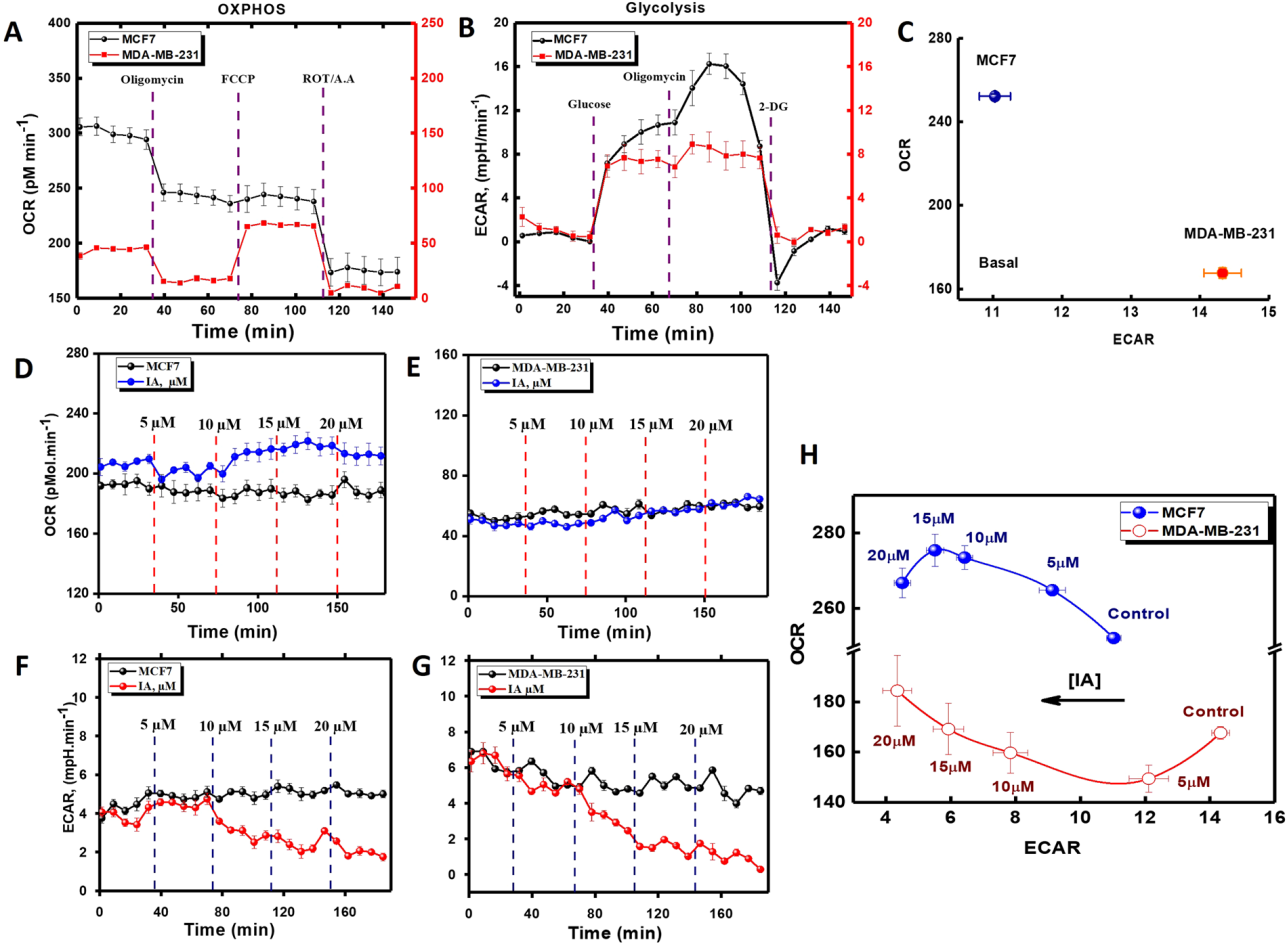
Metabolic phenotyping and effects of instantaneous IA additions on mitochondrial and glycolytic activities in MCF-7 and MDA-MB-231 cells. (**A**,**B**) MCF7 or MDA-MB-231 cells were cultured in XF24-well cell culture microplates (Seahorse Bioscience) at a density of 4 × 10^4^ cells/per well and then incubated for 24 h at 37 °C under 5% CO_2_ atmosphere in proper media. OCR (**A**) and ECAR (**B**) values were normalized to total cell numbers for each cell line in the assay. Data are representative of at least three independent experiments, each with 3–5 statistical replicates and error bars represent SEM. (**C**) Relative ECAR and OCR data from (**A**) and (**B**) were plotted simultaneously to reveal overall relative basal metabolic profiles for each cell model. (**D**,**E**) Instantaneous effects of four successive infusions of 5-µM-IA on basal mitochondrial respiration in MCF7 (**D**) or MDA-MB-231 (**E**) cells. (**F**,**G**) Instantaneous effects of four successive infusions of 5-µM-IA on basal glycolytic activities in MCF7 (**F**) or MDA-MB-231 (**G**) cells. (**H**) Effects of increasing concentration of instantaneously added IA on both OCR and ECAR measures in both cell types.

Breast cancer cell subtypes vary by their source tumor and exhibit highly specific sets of genomic lesions. Such genomic changes are associated with distinct phenotypes leading to a differential response to targeted and untargeted therapies. To explore the functional differences of different breast cell types, we used the Seahorse XF24 Flux Analyzer (Agilent, Germany) to profile oxidative phosphorylation as well as glycolysis in the hormone-responsive MCF7 and the triple-negative MDA-MB-231 cell lines, both accounted as the most commonly used BC cell line models (Fig. 1). Both mitochondrial- and glycolytic-stress assays were carried out using selective substrates/inhibitors of different metabolic states while measuring both oxygen consumption rate “OCR” and extracellular acidification rate “ECAR”. As shown in Fig. 1, relative to MDA-MB-231, MCF7 appears to  relay more on mitochondrial respiration (Fig. 1A,C) and less on glycolysis (Fig. 1B,C) for their bioenergetics demands. We, therefore, moved to test if these metabolic differences render TNBC cells more vulnerable to metabolic draining through glycolytic inhibition by iodoacetate. In Fig. 1(D–H) we show the effects of increasing concentration of instantaneously infused IA on metabolic fluxes in MCF7 and MDA-MB-231 cells. IA caused an immediate and dose-dependent reduction in ECAR in both cells. This was associated with a modest but consistent increase of OCR in MCF7, a trend that was only observed in MDA-MB-231 cells at higher IA concentration; i.e. >15 μM.

### Differential apoptotic responses in iodoacetate-treated cell types

**Figure 2:**
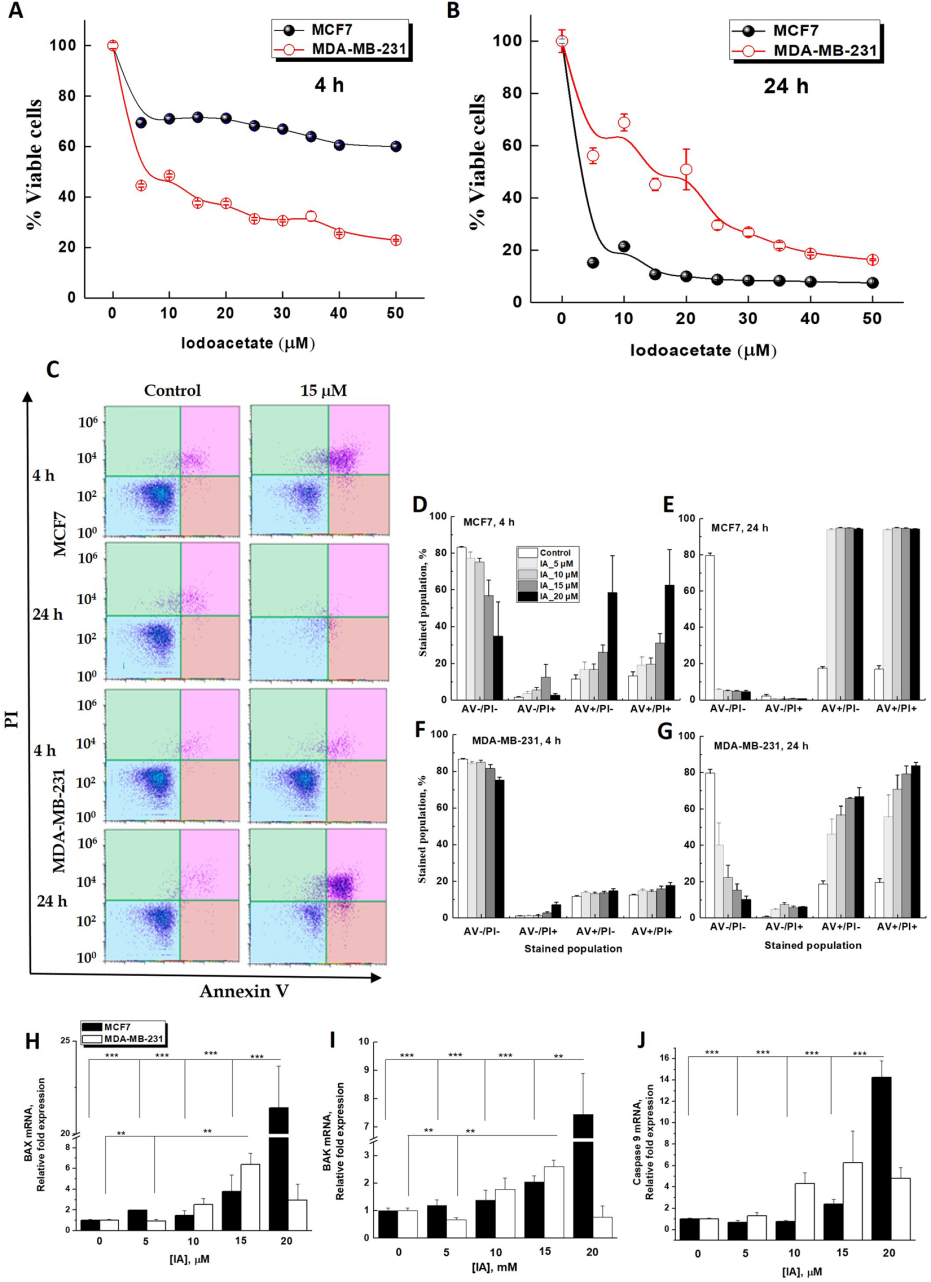
Differential effect of increasing iodoacetate concentration on the viability and apoptotic response of hormone-responsive MCF7 and triple-negative MDA-MB-231 breast cancer cell lines. (**A**,**B**) IA-induced cytotoxicity of MCF7 and MDA-MB-231 was assessed using WST-1 after incubation for 4 h (A) or 24 h (**B**). (**C**) Representative flowcytometric scatter plots of AnnexinV/PI-based detection of early and late apoptosis in MCF7 and MDA-MB-231 following treatments with 0 or 15 µM IA for 4 or 24 h. (**D**–**G**) Quantification of 4 subpopulation AV^−^/PI^−^, AV^−^/PI^+^, AV^+^/PI^−^, AV^+^/PI^+^ in the two cell lines after 4 or 24 h IA treatments. (**H**–**J**) mRNA Relative fold expression changes for both MCF7 and MDA-MB-231 BAX (**H**), BAK (**I**), and Caspase 9 (**J**). Quantification of apoptotic subpopulations was carried out over 3 independent runs where (*) indicates p < 0.05, **p ≤ 0.01, ***p ≤ 0.001.

The above results confirmed that the TNBC cells are more glycolytic than the hormone responsive MCF7 cells and that instantaneous IA treatment selectively inhibited glycolytic and not mitochondrial activities. We then asked if and how long-term glycolytic interruptions may differentially affect survival in hormone-responsive versus triple-negative breast cancer cells. WST-1 cytotoxicity colorimetric assay was used to evaluate the viability of the two BC cell types after 4 and 24 h incubation with increasing iodoacetate concentration (Fig. 2). After 4 h, MDA-MB-231 cells showed greater sensitivity to IA-mediated glycolytic inhibition as ~70% of the cells exhibited low levels of formazan formation, which indicates that only 30% of the cells survived the IA treatment. Meanwhile, losing only 30% of viable cell number, MCF7 cells exhibited more tolerance to glycolytic inhibition by IA (at 4 h, IC_50_ against MCF7 cells >50 μM; IC_50_ against MDA-MB-231 cells = 11.70 ± 0.4 μM, Fig. 2A). However, this trend was completely reversed after 24 h especially at lower IA concentration range 5–30 μM (at 24 h, IC_50_ against MCF7 cells = 4.40 ± 0.1 μM; IC_50_ against MDA-MB-231 cells = 15.40 ± 0.9 μM, Fig. 2B). In tune with clinical characteristics, the cytotoxicity results reveal the ability of the TNBC cells to recover from a cellular insult, in this case mediated by glycolytic disruptions, despite their initial sensitivity to the same treatment relative to the hormone responsive cell type MCF7.

To delineate the mechanism of IA-mediated cell death, we utilized flow cytometry to detect the externalization of phosphatidylserine in apoptotic cells using recombinant annexin V conjugated to green-fluorescent FITC dye and dead cells using propidium iodide (PI). Propidium iodide stains necrotic cells and/or cells in late apoptosis with red fluorescence (AV^−^/PI^+^). Double staining with both probes leaves apoptotic cells with green fluorescence (AV^+^/PI^−^), dead cells with red and green fluorescence (AV^+^/PI^+^), and live cells with little or no fluorescence (AV^−^/PI^−^), Fig. 2C. Following incubations of MCF7 with different iodoacetate concentrations for 4 h, flow cytometric analysis showed a detectable dose-dependent increase in the percentage of apoptotic and dead cells (Fig. 2D). Treatment of MCF7 cells with IA for 24 h induced complete cell death/apoptosis at all concentrations (Fig. 2E). In support of the cytotoxicity results in Fig. 2B, MDA-MB-231 cells were less prone to cell death after 4 h and even with the highest IA dose (20 mM) a resilient sub-population still persisted. Results in Fig. 2D–G were quantified over 3 independent runs.

To further demonstrate the mechanism of IA-induced cell death, q-PCR analysis was employed to determine mRNA levels encoding for BAX, BAK, caspase-3, and caspase-9 proteins in both treated and untreated MCF7 and MDA-MB-231 cells. The initiator caspase-9 activation is related to the mitochondria-mediated intrinsic pathway. Proapoptotic member genes including *Bak*, *Bax* that move from the cytosol to the mitochondrial membrane following apoptotic stimulant. Elevated expression of *Bax* in breast cancer cells boosts sensitivity to apoptotic stimuli and suppresses tumor growth^26^. Expression of both *Bax* and *Bak* genes significantly increased by increasing concentrations of iodoacetate (5, 10, 15, 20 μM) after 24 h incubation (p < 0.05) (Fig. 2H,I). However, this increase was more pronounced in MCF7 cells reaching more than 20 folds for *Bax* and 7 folds for *Bak* at 20 μM iodoacetate. A parallel increase in caspase 9 gene expression was observed only in MCF7 (Fig. 2J), confirming that IA treatment induced apoptotic cell death more effectively in hormone-responsive cells after 24 h. We didn’t detect systematic changes in expression levels of caspase-3 (not shown).

### Differential iodoacetate effects on cell cycle progression and repair mechanism in the two breast cancer cell lines

**Figure 3:**
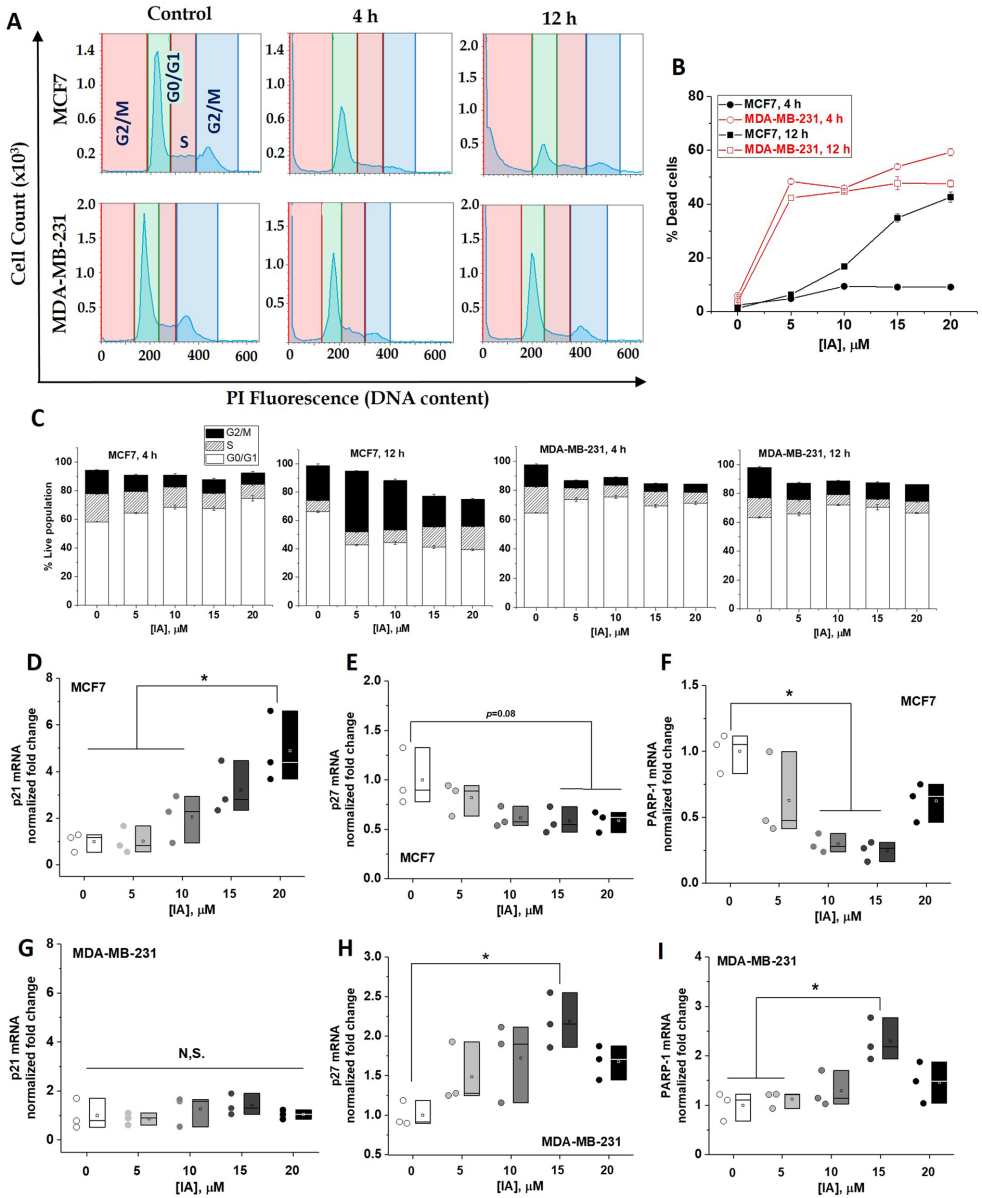
Differential iodoacetate effects on cell cycle progression and repair genes expressions in MCF-7 and MDA-MB-231 cells. (**A**) Representative DNA histograms displaying various cell cycle phases for MCF7 and MDA-MB-231 4 and 12 h after treatment with 15 mM IA. (**B**) Quantification of percentage cell death (sub G1) induced by various [IA] for 4 and 12 h. (**C**) Bar chart representing the percentage of cell population in MCF7 and MDA-MB-231 after IA treatment at (0–20 µM) for 4 and 12 hrs. (**D**–**I**) mRNA expression for both MCF7 (upper row) and MDA-MB-231 (lower row) for p21 (**D**,**G**), p27 (**E**,**H**), and PARP-1 (**F**,**I**). Quantification was carried out over three independent runs. Statistical analyses were carried out using One-way ANOVA where statistical significance was achieved at p < 0.05.

During proliferation, cell cycle checkpoints are set to discover and repair DNA damage. Checkpoints occur late in G1 phase, which allow or delay entry to the S phase. They also occur late in G2 phase which controls entry to mitosis. It is now clear that cell cycle checkpoints are crucial regulators of cell fate, which explains why chemotherapeutic agents are designed to target checkpoints. We thus tested if the observed IA-mediated metabolic disruptions and apoptotic inductions are associated with differential effects on cell cycles in both cell types. Representative time course of the DNA histograms after 4 or 12 h following treatment of MCF7 (upper row, Fig. 3A) or MDA-MB-231 (lower row) with 20 μM IA and quantifications of cell populations at each growth phase treated with different concentrations are shown in Fig. 3(B,C). Under the effect of increasing IA concentration, an overall increase in dead cells is more pronounced in MDA-MB-231 after 4 h (Fig. 3B), which is consistent with the cytotoxicity results in Fig. 2A. However, after 12 h TNBC cells exhibited a lower percentage of dead cells (sub-G1 population) while hormone-responsive cells showed increased numbers of dead cell in proportion with increasing IA concentration. Inspecting the quantifications in Fig. 3C indicates a remarkable growth arrest of IA-treated MCF7 cells in the G2/M and S phases relative to untreated cells, especially after 12 h. However, MDA-MB-231 cells responded differently to IA treatment with slight stall in the G0/G1 phase. These results hint at differential cell death mechanisms induced by glycolytic deprivation in the two cell types. To summarize findings related to cell cycle, recovery of TNBC cells following 12 h of IA treatment was associated with minor growth arrest in G0/G1 phase, whereas hormone responsive cells exhibited a dramatic G2/M and S arrests and this was accompanied by a massive cell death by IA after 12–24 h.

To further explore the impact of iodoacetate on cell cycle progression of breast cancer cells, we assessed its effect on the mRNA expression of the cyclin-dependent kinase inhibitors p21 and p27 proteins that control cell cycle progression and cell death response. We found a significant concentration-dependent increase in p21 (Fig. 3D) and mild decrease in p27 (Fig. 3E) expressions upon IA treatment of MCF7. In contrast, MDA-MB-231 cells exhibited invariant p21 (Fig. 3G) and significantly increased p27 especially at 15 μM IA (Fig. 3H). We also followed the effect of IA on the gene expression profile of poly(ADP-ribose) polymerase-1 (PARP1), an enzyme that plays key roles in genome maintenance, cell death, inflammatory responses, and the control of gene expression programs. PARP1 activity is intimately involved in maintaining genomic stability and its inhibition is currently pursued as an anticancer strategy. Multiple studies have shown that PARP-1 acts as a mediator of cell cycle due to its function as a regulator of various transcriptional factors^27–29^. IA treatment 5–15 μM increased PARP1 expression in MDA-MB-231 cells (Fig. 3I) while decreasing that in MCF7 (Fig. 3F) significantly.

### Glycolytic inhibition by iodoacetate elicits mitochondrial activation only in TNBC cells

**Figure 4:**
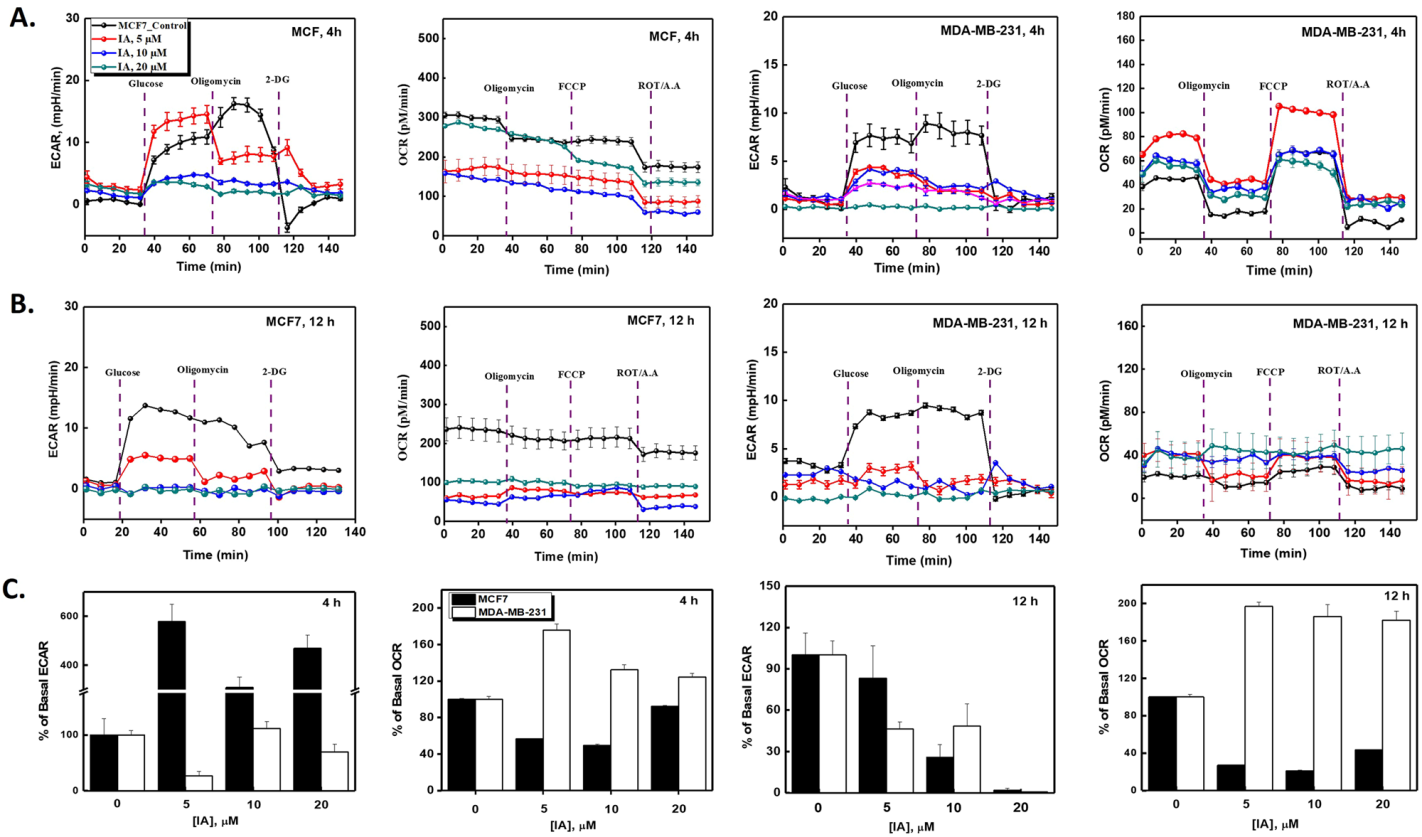
Analyses of differential metabolic effects of IA on bioenergetic parameters in MCF7 and MDA-MB-231 cells. Effects of pre-incubation of the two cell types with 5, 10, or 20 mM IA for 4 **(A)** or 12 h **(B)** on glycolytic as well as mitochondrial bioeneregetic parameters as determined by glycolytic stress tests (probed through ECAR) and mitochondrial stress test (probed through OCR). Results are representative of at least 3 independent runs performed using Seahorse XF metabolic flux analyzer as detailed in the methods section. (**C**) Quantification of basal rates (OCR and ECAR) for MCF7 and MDA-MB-231 pretreated with IA at concentrations of 0, 5, 10, or 20 μM for 4 h or 12 h.

Our results so far reveal that TNBC cells escape long term glycolytic inhibition and show lower signs of apoptotic death, lower expression of apoptotic genes, normal cell cycle regulations, but increase mRNA levels of p27 and PARP1; all compatible with enhanced cell survival. The natural question that arises from these observations is how TNBC cells handle the long-term bioenergetic deprivation induced by IA treatment? We have shown in Fig. 1(D–H) that instantaneous addition of IA disrupts glycolysis completely in both cell types while moderately enhancing mitochondrial oxygen consumption especially in MCF7 cells. This hinted at a potential role for mitochondria in response to glycolytic inhibition. We thus explored how glycolysis and mitochondrial oxidative phosphorylation changed after 4 (Fig. 4A) and 12 h (Fig. 4B) incubations with increasing concentrations of IA. Mean values of parameters pertaining to basal respiration and basal glycolytic activities were quantified and presented in Fig. 4C. It can be readily seen from Fig. 4 that basal glycolytic activity in MCF7 cells did recover remarkably after 4 h-incubation with different IA concentrations and this was associated with ~ 50% reduction in basal mitochondrial respiration. After 12 h, an almost complete metabolic black-out was induced by high IA concentrations in those cells. On the contrary, while IA abolished glycolytic activities mildly after 4 h and drastically after 12 h, mitochondrial activity was remarkably higher in MDA-MB-231 cells. These metabolic adaptations in response to IA treatment appear to be in good agreement with time-dependent cytotoxicity and apoptosis induction results (Figs 1–3).

### Differential role of mitochondria in handling glycolytic deprivation in breast cancer cell types

**Figure 5:**
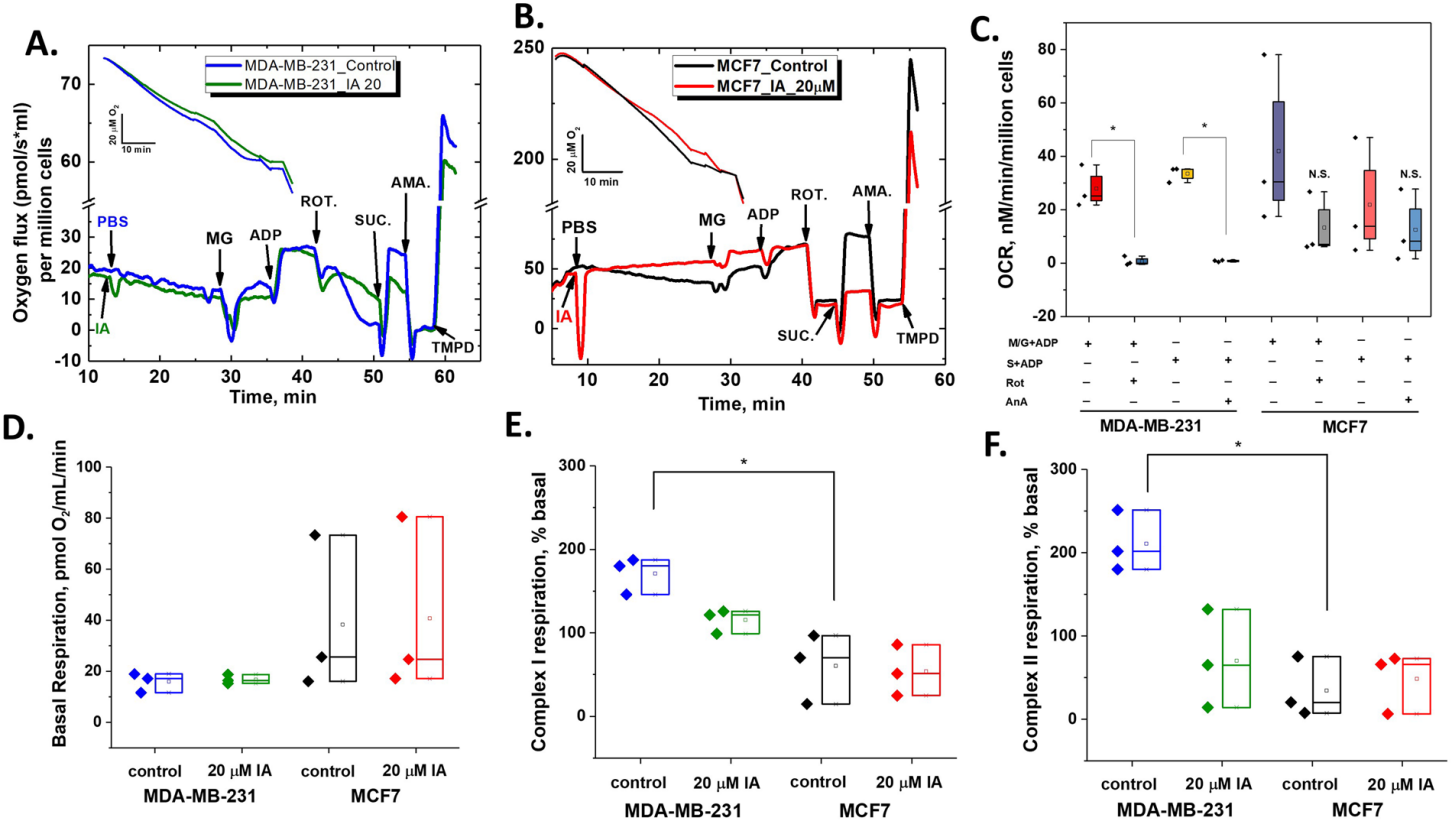
Direct effects of IA on mitochondrial respiratory states of MCF7 and MDA-MB-231 are different. Oxygen level traces (insets) and OCR traces in the absence and presence of 20 mM IA added on saponin-permeabilized cells (4 million cells) measured by Oroboros O2k Oxygraph for both MDA-MB-231 **(A)** and MCF7 **(B)**. Different substrates/inhibitors combinations were sequentially added to obtain standard respiratory parameters pertaining to different electron transport chain complexes in the two cell lines. NADH- and succinate-linked respirations were found to be highly sensitive to complex I inhibition by rotenone and complex III inhibition by antimycin A only in MDA-MB-231 cells **(C)**. While basal respiration was higher in MCF7 cells (**D**), complex I- and complex II- mediated respiration were significantly higher in MDA-MB-231 cells. (**E**,**F**) IA at 20 mM didn’t completely abolish respiratory activities in MDA-MB-231 cells and didn’t affect respiratory activities in MCF7. Quantification was carried out over three independent runs. Statistical analyses were carried out using paired t test where statistical significance was achieved at p < 0.05.

The above results point at the distinctive role for mitochondria in combating bioenergetic stress in TNBC cells following glycolytic inhibition. We hence asked if: (1) individual activities of mitochondrial electron transfer chain complexes (ETC) in the two cells are different; and (2) how ETC complex activities in the two cell types are affected by IA treatment? To approach these questions, we used the Oroboros O2k high-resolution oxygraph (Oroboros Instruments, Austria) to determine substrate-dependent oxygen utilization in permeabilized MCF7 and MDA-MB-231 cells. To allow mitochondrial substrates to access cells freely, saponin was injected and left for 5 minutes to permeabilize plasma membrane before adding the mitochondrial substrates as described in the Materials and Methods section. In a standard experiment, 2.5 million of viable cells were incubated in the two parallel chambers of O2k oxygraph and left for 5 min with saponin to equilibrate in the MiR05 buffer at 37 °C before adding iodoacetate (final concentration 20 µM) and stirring with cells for 15 min. Figure 5(A,B) shows traces for oxygen levels (insets) and OCR calculated in the presence and absence of 20 μM IA for MDA-MB-231 (A) and MCF7 (B). A noticeable difference between the two cell types is the ability of rotenone and antimycin A to completely inhibit (malate + glutamate + ADP)- and (succinate + ADP)-driven oxygen consumptions only in MDA-MB-231 cells (Fig. 5C, N = 3, p < 0.01, by Paired-sample t-test). This may imply that both complex I and complex II are tightly coupled with complex IV of the ETC in those cells. We then compared respiratory parameters including basal- (Fig. 5D), complex I-derived-(Fig. 5E), and complex II-derived- (Fig. 5F) OCRs that were averaged over three independent runs at all conditions. Although basal respiration by MDA-MB-231 cells was generally lower than MCF7 (Fig. 5D), we were puzzled by the finding that respiratory activities mediated by either complex I or II were significantly greater in the former (Fig. 5E,F; N = 3, p < 0.05 by t-test).

**Figure 6:**
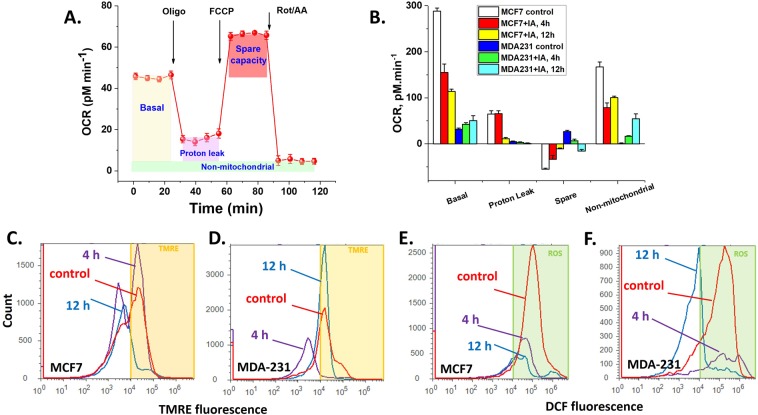
Effects of IA on mitochondrial bioenergetics, transmembrane potential and ROS production. (**A**) The Seahorse mito-stress assay. OCR is measured before and after adding inhibitors/uncoupler to induce different mitochondrial respiratory states (defined in the shaded areas). (**B**) Basal respiration, proton leak, spare capacity, and non-mitochondrial respiratory rates (defined in A) were quantified and compared for both cell types after 4 or 12 h of treatment with 20 mM IA. (**C**,**D**) Effect of IA-treatment time on mitochondrial transmembrane potential as determined by flow cytometric analysis using TMRE staining for both MCF7 and MDA-MB-231 cells; respectively. (**E**,**F**) Flow cytometric analysis of cellular ROS levels using DCF cellular staining of both MCF7 and MDA-MB-231 cells; respectively.

To delineate these paradoxical observations, we analyzed the Seahorse-obtained mitochondrial respiratory profiles in the two cell lines with and without IA treatment (20 μM for 4 and 12 h). Respiratory parameters including: Basal Respiration [OCR^basal^ – OCR^R/A^], ATP-linked respiration [OCR^basal^ – OCR°^ligo^], Proton Leak [OCR^oligo^ – OCR^R/A^], Maximum Respiration [OCR^FCCP^ – OCR^R/A^], Spare Capacity [OCR^FCCP^ – OCR^basal^] were calculated as depicted in Fig. 6A and Basal, Proton Leak, Spare Capacity, as well as Non-mitochondrial oxygen consumption (OCR^R/A^) are quantified over 3 wells and 5 replicas per condition (given in Fig. 6B). These data clearly confirm trends that basal respiratory activity in MCF7 cells are remarkably higher than MDA-MB-231. However, the results reveal that mitochondria of the TNBC MDA-MB-231 cells are more coupled (less proton leak) and possess remarkably greater spare respiratory capacity than hormone responsive MCF7 cells. Moreover, MCF7 cells exhibit rather high non-mitochondrial oxygen consumption. Consequently, although MCF7 cells show significantly higher oxygen consuming activity, most of this activity is non-mitochondrial or due to uncoupled mitochondria. Moreover, the lack of respiratory reserve capacity in MCF7 cells implies that these cells are relatively less competent in handling bioenergetic deprivation.

Extended periods of IA treatments (4–12 h) led to drastic mitochondrial inhibition in MCF7 but moderately activated mitochondrial basal respiration in MDA-MB-231 cells (Fig. 6B). To confirm this observation we assessed the effect of IA on mitochondrial transmembrane potential “ΔΨ_m_” using cell staining with Tetramethylrhodamine ethyl ester perchlorate (TMRE) as analyzed by flow cytometry. Again, IA treatment caused a gradual loss of mitochondrial transmembrane potential in MCF7 cells (Fig. 6C) while a clear recovery after 12 h is seen with MDA-MB-231 cells (Fig. 6D). Since mitochondrial activities are tightly connected with levels of reactive oxygen species (ROS), which is believed in turn to contribute to apoptosis induction and cell cycle arrest, we also explored if IA induces differential changes in levels of ROS in the two different cell lines. We measured intracellular ROS-mediated oxidation of the fluorescent dye, 2′,7′-dichlorodihydrofluorescein diacetate (H2DCF-DA) by flow cytometry. We found that treatment of MCF7 and MDA-MB-231 cells with iodoacetate for 4 or 12 h induced measurable decrease of ROS levels in a concentration-dependent manner (5–20 µM) relative to control untreated cells. This result role out ROS as a major factor in the induction of cell death by IA in both cell types.

### Glycolytic inhibition by iodoacetate completely eradicates MCF7 cells while eliciting a resilient MDA-MB-231 subpopulation

**Figure 7:**
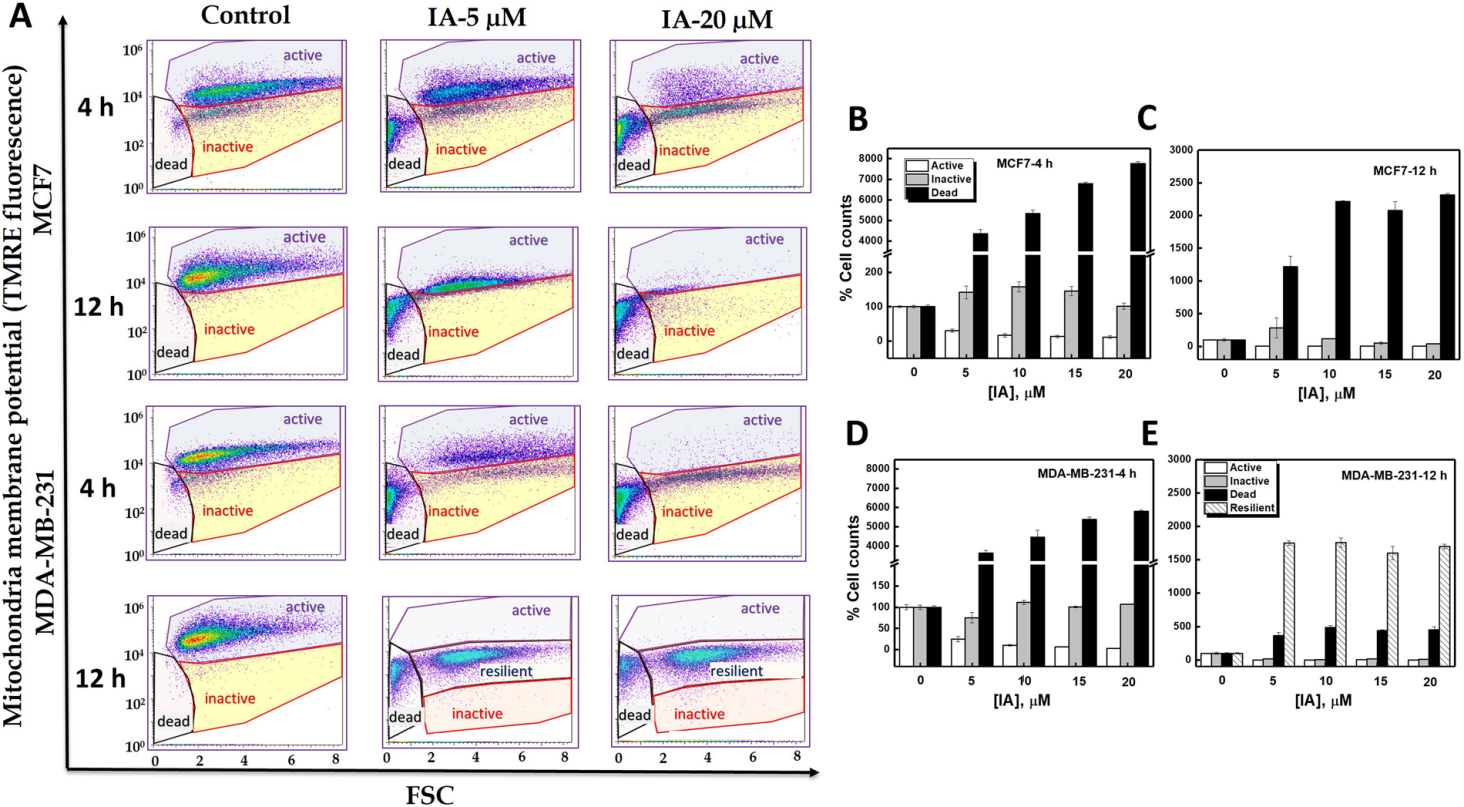
Analysis of mitochondrial response to glycolytic inhibition by IA by flowcytometry reveals the revival of a resilient subpoulation of metabolically active MDA-MB-231 but not MCF7 cells. (**A**) Flow cytometric analysis for mitochondrial membrane potential using TMRE cellular staining of both MCF7 and MDA-MB-231 cells after 4 or 12 h of IA treatment at (0, 5, 20 µM). TMRE staining was carried out to detect mitochondrial membrane depolarization status in IA-treated breast cancer cells. 4 or 12 h post treatments, cells were harvested and stained with 100 nM TMRE as described in Material and Methods. Greater uptake of TMRE indicates more polarized mitochondria which is reflected in higher fluorescence values. Cells exhibited distinct populations that were all sensitive to IA treatment dose and time (quantified in **B**–**E**). Data are representative of three independent experiments with two replicates and all [IA]-related changes were found to be statistically significant when compared with controls by ANOVA.

The results in Fig. 6C,D incited us to systematically analyze the effects of IA concentration and exposure time on mitochondria within cellular populations using TMRE fluorescence assessments by flow cytometry. Similar to previous reports^30^, light scatter analysis revealed that cell subpopulations that were negative or dim for TMRE staining produced a low forward scatter (FSC) signal, whereas viable cell populations brightly stained with mitochondrial dyes produced a high FSC signal (Fig. 7A). Using this analysis, we detected distinct cellular subpopulations with metabolically active (high TMRE fluorescence, high FSC signal) or inactive mitochondria (low TMRE fluorescence, high FSC signal) in addition to dead cells with low FSC signal and TMRE staining. We followed the dependence of these subpopulations on IA concentration and exposure time in the two cell types. After 4 h, and upon increasing the IA dose (5–20 μM) both cell types exhibited increased proportions of metabolically inactive and dead cell populations on the account of active cell populations (Fig. 7A,B,D). After 12 h, while MCF7 showed only dead cells remaining (Fig. 7C), MDA-MB-231 exhibited a new population with moderately high TMRE fluorescence and high FSC signal (resilient cell-population, Fig. 7E).

## Discussion

TNBCs constitute about 15–20% of all BCs and is clinically characterized by shorter overall survival and an early peak of distant recurrences at 3 years after diagnosis^3^. Epidemiologic studies revealed a high prevalence of triple-negative breast cancers in younger women and those of African descent^31^. TNBC has an aggressive clinical behavior, with a higher risk of both local and distant relapses that frequently manifest as visceral and/or brain metastases. Following initial diagnosis, TNBC is associated with poor survival after recurrence mainly due to the lack of effective therapies in general for this aggressive subtype of breast cancer^32^. Tailored therapies, such as hormonal and anti-HER2 therapies, are not applicable for the lack of target receptors. Preclinical and clinical studies have suggested that TNBC subtypes maybe targeted by DNA-damaging agents and PARP inhibitors for those harboring BRCA1 dysfunction^33^. It was recently concluded that PARP inhibitors are promising against TNBC but the field would greatly benefit from the identification of biomarkers to predict treatment response and strategies to counteract emerging resistance^34^. Understanding the underlying molecular pathways driving the aggressive behavior of TNBC is crucial to identify molecular targets for better systemic treatment options^2^.

A conceptual framework that is emerging implicates metabolism and mitochondria as pivotal players in cancer biology including development and progression, metabolic reprogramming, acquisition of metastatic characteristics, and response to chemotherapeutic agents^35^. Due to cancer-associated down-regulated and dysfunctional mitochondria, aerobic glycolysis is suggested to fulfill bioenergetic demands that drive cancer progression, growth, survival, metastasis, immune evasion, resistance to treatment, and disease recurrence^9,11^. Targeting glycolysis is recently presented as a viable strategy to combat a broad range of cancers^36–40^. In fact, targeting metabolism and mitochondria are gaining momentum not only as a new therapeutic strategy, but also as tools to understand cancer etiology and progression. Along this logic, we asked if differences in metabolic machineries between a hormone responsive cell line (MCF7) and a TNBC cell line (MDA-MB-231) may shed light on oncologic differences between the two original cancer subtypes especially in response to a potent cytotoxic agent.

We investigated metabolic modulations and differential anti-cancer effects induced by iodoacetate treatment of MCF7 (invasive, metastatic, and hormone-responsive cells) versus MDA-MB-231 (invasive, metastatic, and TNBC cells). Iodoacetate is an alkylating agent with antineoplastic effect and is reported as a glycolytic inhibitor through GAPDH modulation^41^. Our results demonstrated that iodoacetate induces metabolic shifts from glycolysis to oxidative phosphorylation “OXPHOS” in a dose and time-dependent manner albeit differently in the two BC cell lines. Upon infusion, IA was found to completely inhibit glycolytic activities in both cell types but more effectively in MDA-MB-231. In addition to instantaneous effects of IA, we explored short-term (4 h), and long-term (12 h) metabolic modulations elicited by various IA concentrations. After 4 h incubation with the drug, MCF7 cells exhibited a dramatically enhanced basal glycolysis and reduced OXPHOS which apparently allowed these cells to survive IA-induced cytotoxicity. However, these cells were completely dead after 12 h as both mitochondria and glycolysis were drained. On the other hand, and after an early wave of cell death, MDA-MB-231 cells handled glycolytic deprivation through mitochondrial activation, which induced the growth of a secondary cell population after 12 h incubation with IA. In a sense, this resembles the situation *in vivo* where TNBC patients are known to respond to chemotherapy but suffer from ill clinical outcomes including early relapse and metastatic spread^42,43^.

To add insights into the underlying molecular mechanisms of IA-induced cytotoxicity, we studied cell cycle, apoptosis markers, ROS levels, and mitochondrial activity with and without IA treatments of the two cell lines. IA treatment was found to induce early and late apoptotic cell death, growth arrest in the G2/M and S phases of the cell cycle, and dramatically increase pro-apoptotic genes’ expression in MCF-7 but for a lesser extent in MDA-MB-231 cells. Specifically, we found that in MCF7 breast cancer cells, iodoacetate mediates apoptosis by upregulating *BAX/BAK* gene expression which in turn induced caspase 9 and p21 activation leading to increased cytotoxicity as confirmed by flow cytometry multiple measures. Cell cycle regulators p21 and p27 are members in a family of critical multifaceted inhibitors of cyclin-dependent kinases and thus act as important checkpoints regulators. Their down-regulation or loss of function were found to associate with increased prevalence of many human malignancies^44^. In response to IA insult, MCF7 cells showed significantly increased normalized level of p21 mRNA which is expected to inhibit proliferation^45^. P27 is more dynamic and was reported to appear transiently in the cytoplasm during the G1-S transition before it degrades^46^. This may explain the lack of similarity in the expression profiles of p21 and p27 following IA treatment in MCF7 cells. However, no consistent changes in the normalized levels of p21 mRNA in MDA-MB-231 cells were observed in response to IA treatment, which agrees with the observed lack of impact on cell cycle, especially after 12 h. Nevertheless, we observed a significant increase in the normalized level of p27 in an IA-dose dependent manner.

We also followed the effect of IA treatment on the mRNA expression level of PARP-1, which is a cell nuclear enzyme involved in DNA repair, maintenance of genome integrity, and in post-translational ribosylation of proteins^47^. In response to death signals, PARP was found to specifically cleave during apoptosis in several model systems, which presumably acts as a backup system to maintain the genome^48^. Interestingly, while IA-treatment up to 15 μM reduced normalized PARP-1 level in MCF7 cells, it significantly increased it in MDA-MB-231 cells. In fact, expression profiles of p27 and PARP1 are similar within each cell line but follow opposite trends when the two cell lines are compared. This highlights the association between these two factors especially in a p53 mutant cells such as MDA-MB-231^49^. It was reported that increasing p27 stability permits cells to survive metabolic stress through the avoidance of apoptosis and the activation of autophagy^50^. Moreover, preservation of mitochondrial membrane potential leading to enhanced cell survival under oxidative stress conditions was found to critically depend on PARP-1 activity^51^. We suggest that the differential increase in *PARP-1* and *p27* expressions in MDA-MB-231 cells is related to the enhanced cell revitalization following glycolytic disruption and DNA damage inflected by IA.

**Figure 8:**
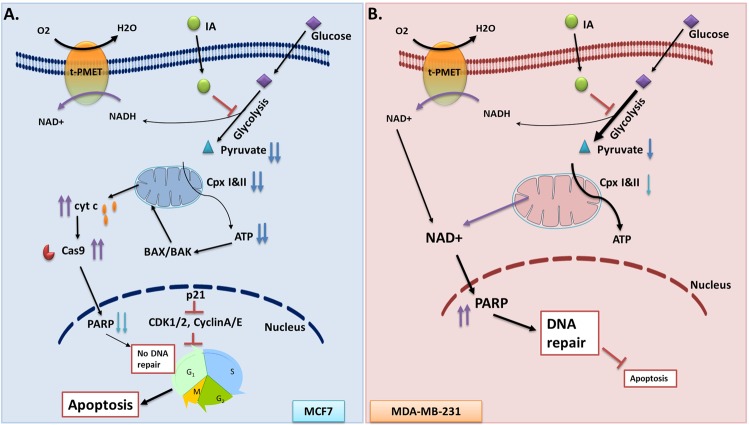
Schematic representation of the mechanism of IA-induced cell death in MCF7 cells and the role of mitochondria in rescuing MDA-MB-231 cells under the same treatment. Our analyses showed that basal respiratory activity in MCF7 cells were remarkably higher than MDA-MB-231 while the later showed higher glycolytic activities. However, multiple converging evidence from the current work revealed that mitochondria of MDA-MB-231 cells were more coupled (less proton leak) and possess remarkably greater spare respiratory capacity than hormone responsive MCF7 cells. Moreover, MCF7 cells exhibit significantly higher non-mitochondrial oxygen consumption which might be attributable to cell surface oxygen consumption as mediated by trans-plasma membrane electron-transport (t-PMET). It is conceivable that increased cellular levels of NAD^+^ either through enhanced mitochondrial and/or t-PMET activities may explain increased PARP-1 levels leading to augmented DNA repair mechanism in treated TNBC cells. Moreover, the lack of respiratory reserve capacity in MCF7 cells implies that these cells are relatively less competent in handling bioenergetic stress which is reflected in increased apoptosis, lowered DNA repair, and cell cycle arrest leading to effective cell death.

The interplay between the repair mechanism and nicotinamide adenine dinucleotide (NAD^+^)-dependent metabolic switch was suggested^52^. This along with our results provided impetus to explore how mitochondria contribute to the ability of TNBC cells to avoid IA-induced cell death and to provide alternative bioenergetics sources in TNBC cells that are originally characterized with high glycolytic activities^12^. We thus analyzed mitochondrial respiratory function in depth using both of the Seahorse and Oroboros metabolic analyzers. We concluded that basal respiratory activity in MCF7 cells were remarkably higher than MDA-MB-231. However, multiple converging evidence revealed that mitochondria of MDA-MB-231 cells were more coupled (less proton leak) and possess remarkably greater spare respiratory capacity than hormone responsive MCF7 cells. Moreover, MCF7 cells showed significantly higher non-mitochondrial oxygen consumption. Consequently, although MCF7 cells show dramatically higher oxygen consuming activity, most of this activity is non-mitochondrial or due to uncoupled mitochondria. Moreover, the lack of respiratory reserve capacity in MCF7 cells implies that these cells are relatively less competent in handling bioenergetic stress. Indeed, IA treatment remarkably reduced mitochondrial respiratory activities after 4 h and even more after 12 h in MCF7. Meanwhile, both basal and non-mitochondrial oxygen consumptions in MDA-MB-231 were increased by IA on the account of these cells’ respiratory spare (Fig. 6B). Non-mitochondrial oxygen consumption (cell surface oxygen consumption) as mediated by trans-plasma membrane electron-transport (t-PMET) is documented in glycolytic breast cancer such as TNBCs and was suggested to support glycolytic energy metabolism by re-oxidizing NADH to NAD^+^^53^. It is conceivable that increased cellular levels of NAD^+^ either through enhanced mitochondrial and/or t-PMET activities may explain increased PARP-1 levels leading to augmented DNA repair mechanism in treated TNBC cells (Fig. 8). PARP1 is a key cellular regulator with increasingly recognized critical roles in cellular energetics and death signaling, allowing activity of this protein to serve as a switch between cell fates and to affect both tumor proliferation and therapeutic response. Meanwhile, inhibition of PARP1 was reported to enhance the expression of checkpoint proteins, including p21 and p27^29^. This led us to propose the centrality of PARP1 in the TNBC cellular response facing glycolytic blockade.

From a clinical perspective, there is a critical need to understand not only how metabolism echoes oncologic transformations, but also how it dictates tumor heterogeneities, interaction with the microenvironment, metastasis, and response to therapies. Such knowledge would enable the identification of synergistic therapeutic approaches exploiting metabolic pathways as potential targets for synthetic lethality. Although metabolic reprogramming is increasingly recognized as promising therapeutic windows, attempts to target the enhanced glycolytic activity in cancer; e.g. using 2-deoxyglucose were not fruitful so far (reviewed in^54,55^). Indeed, the current study provides additional evidence that targeting glycolysis might not be an effective strategy in the case of TNBC and suggests that the mitochondrial aid-in-reserve must be selectively blocked. Interestingly, it was found that loss of p53 sensitizes cells to metformin, which enhances the use of fatty acids for energy production and triggers autophagy through the inhibition of mitochondrial complex I and activation of AMPK^56^. Both fatty acid metabolism and autophagy are known to promote cell survival under conditions of scarce nutrients, but a functional p53 is essential for both processes. Since the p53 gene was found to be mutated in approximately 80% of basal/TNBC^57^, one may suggest here that combining PARP1 inhibitors with metformin may provide a viable therapeutic approach for specific synthetic lethality in TNBC.

Mitochondria are recognized targets for treating aggressive, metastatic, and chemoresistant tumors [reviewed recently in^58–60^]. However, it is worthy to mention that most of these studies are carried out in the realm of basic cancer research and preclinical domains. The fact that *in vitro* cancer cells behave remarkably different as compared with tumor microenvironments necessitates extensive studies to understand tumor heterogeneities, and to identify suitable molecular markers for types of stroma in order to predict treatment response *in vivo*. For example, an interesting recent report linked elevated aldo-keto reductase AKR1B10 expression in ER^−^ and HER2^+^ breast cancers with an increased incidence of metastatic relapse at secondary sites that was associated with increased fatty acid utilization^61^. These authors determined that breast cancer cells with high AKR1B10 expression do not display altered survival or proliferation properties when cultured *in vitro* or when grown as primary tumors in the fat pads of recipient mice but are more robust when cultured in nutrient poor conditions or when colonizing the lungs. It remains to be answered if mitochondria-mediated metabolic adaptations in transforming cancer stem cells, metastasizing tumors, and tumor subtypes share targetable similarities that are distinguishable from proliferating normal cells.

In summary, although described often as glycolytic cancer cells, TNBC cells possess tightly coupled mitochondria with measurable respiratory reserve capacity. On the other hand, MCF7 showed remarkable oxygen-consuming activities, but our analysis indicated that most of these activities are due to uncoupled mitochondria or non-mitochondrial in origin. Cytotoxic effects of iodoacetate in MCF7 and MDA-MB-231 human breast cancer cells are mediated by different cellular mechanisms. Whereas glycolytic disruption by IA appears effective in both cell lines, the resultant cascades of molecular events triggered massive apoptotic response in the hormone sensitive MCF7 cells only. We provided converging evidence that mitochondrial activation in a subpopulation of MDA-MB-231 cells mediates the survival of these cells and their subsequent proliferation under metabolic stress and DNA damage by IA. It is therefore conceivable that mitochondria play pivotal roles in early relapse and metastatic spread of TNBC and thus provide an additional target for combined therapies against this devastating disease.

## Data Availability

All materials, data and associated protocols are available to readers upon reasonable requests from the corresponding author.

## References

[1] Global Burden of Disease Cancer, C. (2017). Global, Regional, and National Cancer Incidence, Mortality, Years of Life Lost, Years Lived With Disability, and Disability-Adjusted Life-years for 32 Cancer Groups, 1990 to 2015: A Systematic Analysis for the Global Burden of Disease Study. JAMA oncology.

[2] Collignon J, Lousberg L, Schroeder H, Jerusalem G (2016). Triple-negative breast cancer: treatment challenges and solutions. Breast cancer.

[3] Dent R (2007). Triple-negative breast cancer: clinical features and patterns of recurrence. Clinical cancer research: an official journal of the American Association for Cancer Research.

[4] Liedtke C (2008). Response to neoadjuvant therapy and long-term survival in patients with triple-negative breast cancer. Journal of clinical oncology: official journal of the American Society of Clinical Oncology.

[5] Schulze A, Harris AL (2012). How cancer metabolism is tuned for proliferation and vulnerable to disruption. Nature.

[6] Tasselli L, Chua KF (2012). Cancer: Metabolism in ‘the driver’s seat. Nature.

[7] Wellberg EA (2016). The glucose transporter GLUT1 is required for ErbB2-induced mammary tumorigenesis. Breast cancer research: BCR.

[8] Shen F, Li J, Zhu Y, Wang Z (2016). Systematic investigation of metabolic reprogramming in different cancers based on tissue-specific metabolic models. Journal of bioinformatics and computational biology.

[9] Vander Heiden MG, Cantley LC, Thompson CB (2009). Understanding the Warburg effect: the metabolic requirements of cell proliferation. Science.

[10] DeBerardinis RJ, Lum JJ, Hatzivassiliou G, Thompson CB (2008). The biology of cancer: metabolic reprogramming fuels cell growth and proliferation. Cell metabolism.

[11] Yeung SJ, Pan J, Lee MH (2008). Roles of p53, MYC and HIF-1 in regulating glycolysis - the seventh hallmark of cancer. Cellular and molecular life sciences: CMLS.

[12] Pelicano H (2014). Mitochondrial dysfunction in some triple-negative breast cancer cell lines: role of mTOR pathway and therapeutic potential. Breast cancer research: BCR.

[13] Zhang JY (2015). Critical protein GAPDH and its regulatory mechanisms in cancer cells. Cancer biology & medicine.

[14] Hildebrandt T, Knuesting J, Berndt C, Morgan B, Scheibe R (2015). Cytosolic thiol switches regulating basic cellular functions: GAPDH as an information hub?. Biological chemistry.

[15] Cerella C, Dicato M, Diederich M (2014). Modulatory roles of glycolytic enzymes in cell death. Biochemical pharmacology.

[16] QUASTEL, I. J. B. J. H. Effects of Metabolic Inhibitors on Energy Metabolism of Ehrlich Ascites Carcinoma Cells. *Nature*, 44–46 (02 January 1965). 10.1038/205044a0 14283130

[17] Doherty JR, Cleveland JL (2013). Targeting lactate metabolism for cancer therapeutics. The Journal of clinical investigation.

[18] Apffel CA, Arnason BG, Peters JH (1966). Induction of tumour immunity with tumour cells treated with iodoacetate. Nature.

[19] Ra, H. Carbohydrate metabolism: Textbook of Biochemistry, Chap 7, 4th ed. 293–294 (1992).

[20] Sperling O, Bromberg Y, Oelsner H, Zoref-Shani E (2003). Reactive oxygen species play an important role in iodoacetate-induced neurotoxicity in primary rat neuronal cultures and in differentiated PC12 cells. Neuroscience letters.

[21] Badwey JA, Karnovsky ML (1980). Active oxygen species and the functions of phagocytic leukocytes. Annual review of biochemistry.

[22] Li C (2017). A ROR1-HER3-lncRNA signalling axis modulates the Hippo-YAP pathway to regulate bone metastasis. Nature cell biology.

[23] Cepeda MA, Evered CL, Pelling JJH, Damjanovski S (2017). Inhibition of MT1-MMP proteolytic function and ERK1/2 signalling influences cell migration and invasion through changes in MMP-2 and MMP-9 levels. Journal of cell communication and signaling.

[24] Marrache S, Dhar S (2015). The energy blocker inside the power house: Mitochondria targeted delivery of 3-bromopyruvate. Chemical science.

[25] Pike Winer LS, Wu M (2014). Rapid analysis of glycolytic and oxidative substrate flux of cancer cells in a microplate. PloS one.

[26] Bargou RC (1996). Overexpression of the death-promoting gene bax-alpha which is downregulated in breast cancer restores sensitivity to different apoptotic stimuli and reduces tumor growth in SCID mice. The Journal of clinical investigation.

[27] Simbulan-Rosenthal CM (2003). PARP-1 binds E2F-1 independently of its DNA binding and catalytic domains, and acts as a novel coactivator of E2F-1-mediated transcription during re-entry of quiescent cells into S phase. Oncogene.

[28] Carbone M (2008). Poly(ADP-ribosyl)ation is implicated in the G0-G1 transition of resting cells. Oncogene.

[29] Yang L (2013). Identification of poly(ADP-ribose) polymerase-1 as a cell cycle regulator through modulating Sp1 mediated transcription in human hepatoma cells. PloS one.

[30] Barteneva NS, Ponomarev ED, Tsytsykova A, Armant M, Vorobjev IA (2014). Mitochondrial staining allows robust elimination of apoptotic and damaged cells during cell sorting. The journal of histochemistry and cytochemistry: official journal of the Histochemistry Society.

[31] Anders C, Carey LA (2008). Understanding and treating triple-negative breast cancer. Oncology.

[32] Lin NU (2008). Sites of distant recurrence and clinical outcomes in patients with metastatic triple-negative breast cancer: high incidence of central nervous system metastases. Cancer.

[33] Kurebayashi J (2009). Possible treatment strategies for triple-negative breast cancer on the basis of molecular characteristics. Breast cancer.

[34] Robert Marie, Patsouris Anne, Frenel Jean-Sébastien, Gourmelon Carole, Augereau Paule, Campone Mario (2018). Emerging PARP inhibitors for treating breast cancer. Expert Opinion on Emerging Drugs.

[35] Giuseppe Gasparre RR (2017). Pierre Sonveaux. Mitochondria in Cancer..

[36] Kremer JC (2017). Arginine Deprivation Inhibits the Warburg Effect and Upregulates Glutamine Anaplerosis and Serine Biosynthesis in ASS1-Deficient. Cancers. Cell reports.

[37] Li HM (2017). Blockage of glycolysis by targeting PFKFB3 suppresses tumor growth and metastasis in head and neck squamous cell carcinoma. Journal of experimental & clinical cancer research: CR.

[38] Han RL, Wang FP, Zhang PA, Zhou XY, Li Y (2017). miR-383 inhibits ovarian cancer cell proliferation, invasion and aerobic glycolysis by targeting LDHA. Neoplasma.

[39] Zhu W (2017). MicroRNA-98 Suppress Warburg Effect by Targeting HK2 in Colon Cancer Cells. Digestive diseases and sciences.

[40] Li W (2016). Resveratrol inhibits Hexokinases II mediated glycolysis in non-small cell lung cancer via targeting Akt signaling pathway. Experimental cell research.

[41] Bhardwaj V, Rizvi N, Lai MB, Lai JC, Bhushan A (2010). Glycolytic enzyme inhibitors affect pancreatic cancer survival by modulating its signaling and energetics. Anticancer research.

[42] Malorni L (2012). Clinical and biologic features of triple-negative breast cancers in a large cohort of patients with long-term follow-up. Breast cancer research and treatment.

[43] Kemme HN, Kuijpers W (1966). Delayed type allergic reactions of the lower respiratory tract. An experimental study in guinea-pigs. Practica oto-rhino-laryngologica.

[44] Abukhdeir AM, Park BH (2008). P21 and p27: roles in carcinogenesis and drug resistance. Expert reviews in molecular medicine.

[45] Xiong Y (1993). p21 is a universal inhibitor of cyclin kinases. Nature.

[46] Connor MK (2003). CRM1/Ran-mediated nuclear export of p27(Kip1) involves a nuclear export signal and links p27 export and proteolysis. Molecular biology of the cell.

[47] Nunez G, Benedict MA, Hu Y, Inohara N (1998). Caspases: the proteases of the apoptotic pathway. Oncogene.

[48] Okuma HS, Yonemori K (2017). BRCA Gene Mutations and Poly(ADP-Ribose) Polymerase Inhibitors in Triple-Negative Breast Cancer. Advances in experimental medicine and biology.

[49] Mizuno S (2009). Hypoxia regulates human lung fibroblast proliferation via p53-dependent and -independent pathways. Respiratory research.

[50] Liang J (2007). The energy sensing LKB1-AMPK pathway regulates p27(kip1) phosphorylation mediating the decision to enter autophagy or apoptosis. Nature cell biology.

[51] Tapodi A (2005). Pivotal role of Akt activation in mitochondrial protection and cell survival by poly(ADP-ribose)polymerase-1 inhibition in oxidative stress. The Journal of biological chemistry.

[52] Li D (2014). BRCA1 as a nicotinamide adenine dinucleotide (NAD)-dependent metabolic switch in ovarian cancer. Cell cycle.

[53] Herst PM, Berridge MV (2007). Cell surface oxygen consumption: a major contributor to cellular oxygen consumption in glycolytic cancer cell lines. Biochimica et biophysica acta.

[54] Vander Heiden MG (2011). Targeting cancer metabolism: a therapeutic window opens. Nature reviews. Drug discovery.

[55] Porporato PE, Dhup S, Dadhich RK, Copetti T, Sonveaux P (2011). Anticancer targets in the glycolytic metabolism of tumors: a comprehensive review. Frontiers in pharmacology.

[56] Buzzai M (2007). Systemic treatment with the antidiabetic drug metformin selectively impairs p53-deficient tumor cell growth. Cancer research.

[57] Synnott NC (2017). Mutant p53: a novel target for the treatment of patients with triple-negative breast cancer?. International journal of cancer.

[58] Emmings Edith, Mullany Sally, Chang Zenas, Landen Charles N., Linder Stig, Bazzaro Martina (2019). Targeting Mitochondria for Treatment of Chemoresistant Ovarian Cancer. International Journal of Molecular Sciences.

[59] Bosc C, Selak MA, Sarry JE (2017). Resistance Is Futile: Targeting Mitochondrial Energetics and Metabolism to Overcome Drug Resistance in Cancer Treatment. Cell metabolism.

[60] Guerra F, Arbini AA, Moro L (2017). Mitochondria and cancer chemoresistance. Biochimica et biophysica acta. Bioenergetics.

[61] van Weverwijk A (2019). Metabolic adaptability in metastatic breast cancer by AKR1B10-dependent balancing of glycolysis and fatty acid oxidation. Nature communications.

